# Pyorrhea Alveolaris, from the Standpoint of the General Practitioner

**Published:** 1913-08-15

**Authors:** A. J. Cottrell

**Affiliations:** Knoxville


### PYORRHEA ALVEOLARIS, FROM THE STANDPOINT OF THE GENERAL PRACTITIONER.

BY DR. A. J. COTTRELL, OF KNOXVILLE.

Read June 15, 1913, before the Tennessee State Dental Association.

It is not the purpose of this paper to enter into an exhaustive study of what is generally termed Pyorrhea Alveolaris, or to advance any new ideas in regard to its etiology or treatment, but to give expression to some conclusions based on several years clinical observations, particularly for the benefit of those who are not in the habit of treating this disease.

Let me say in the beginning, that regardless of what the belief of the individual may be in regard to Pyorrhea as a proper field for the specialist, the fact remains that, as far as its treatment and cure is concerned, the great bulk of the work that is done in this line must still be performed by the general dental practitioner, and that as far as the local treatment is concerned, there is nothing that is known about it, nor any thing that can be done for its amelioration that can not be known and should not be done by any capable general practitioner. I mention this because so much has been said along this line that is misleading, and I think that the general practitioner who feels that his skill and knowledge is such that he must refer his pyorrhea cases to some one else, will soon have nothing left for himself but amalgam fillings and rubber plates.

Our nomenclature for the pathological conditions of the gums and alveolar process is very deficient, as it has come to be that all, or nearly all inflammations of these structures of chronic nature are classed as Riggs Disease, when the truth is that many of these conditions are simply due to a lack of care, and the only treatment needed is simply to clean the teeth and keep them clean. Hence the easy manner in which many speak of "curing pyorrhea."

Much has been said and written as to the cause of the disease, some claiming that it is simply and purely local, Dr. Patterson, of Kansas City, takes this position and defends it well; Dr. Talbot, of Chicago, is the original Champion of the constitutional theory, claiming for it a deep seated predisposing cause, the result of defective elimination, and placing it in the same class with rheumatism, gout, diabetes, etc. Both are right, and the concensus of opinion favors Dr. Talbot in his belief as to the constitutional origin of the disease, holding that the local conditions, of course, intensify, or we might say, precipitate the trouble.

There are many cases where the cause is purely local; notably the lack of contact between adjoining teeth usually the result of badly contoured fillings, permitting the constant impaction of the food against the gum tissue in the interdental spaces, the loss of teeth, permitting the tipping and shifting of adjoining teeth, which seems often to break down the gum and alveolar attachment on the surfaces toward the space left by extraction. These conditions are present so very frequently that they should be borne in mind when filling compound approximal cavities or before deciding on extraction.

Badly fitting shell crowns are often mentioned as a cause of this kind of trouble, and are certainly productive of some very bad results, but it is my opinion that crowns of any kind as a cause of pyorrheal pockets are almost negligible. I have seen plenty of them that have kept up a chronic state of inflammation of the gum margins, but very few that had pockets about them, or that had pockets that seemed to be caused by the crowns.

But whatever the cause, and whatever the different opinions as to the cause, there is one thing upon which all are agreed—there can not be a cure or an appreciable amelioration, without *efficient* and *sufficient* local treatment, and it does not matter what future developments may bring, the dental practitioners services must always contribute to the cure of pyorrhea.

A very interesting fact in the study of the clinical features of pyorrhea is the variety of manifestations, and they seem to be different in different localities. I would mention, in passing, that each different manifestation seems to have a different prognosis.

The popular conception of pyorrhea is that it is always accompanied by a flow of pus from the pockets. Such is not the case, and it has been my experience that the most obstinate cases and those which have the most unfavorable prognosis, are those in which no pus is perceptible. Again there are many cases where no calcareous deposits of any kind are to be encountered. Others still where there is no turgid condition of the gum margins, and yet the pockets extend almost to the extremity of the roots. Then there are those where neither pus, deposits nor inflammation are present, yet the gums and alveolar processes seem to be gradually losing their integrity and shrinking away, causing the teeth to loosen as completely as if the most violent inflammatory conditions were present. These are the cases which seem to be of purely constitutional origin, and are the ones that put me "up in the air" completely.

According to my observation when pus is present it is usual that there is only one pocket, more often on the labial or lingual aspect of the root and extending perhaps to the mesial or distal surface, with the balance of the attachment practically normal. Of course, there are many variations, but this is the usual manifestation. Now in the minds of most practitioners if there is no pus the situation is not serious, but to my mind just the reverse is the case, for two or three reasons. First, we find that thorough scaling and curetting will usually cause the cessation of the flow of pus and bring on the healing of the gum tissue; another is, that when it does heal the gum usually shrinks to the point of attachment of the periosteum, leaving no open space between gum and tooth for the collection of debirs and the formation of bacterial plaques. Again, there is no deception about these cures, as long as the pus continues, we *know* that our work has not been complete and there is yet something lacking.

Another peculiar feature is the number of cases which present themselves in which only certain surfaces of certain teeth are affected, sometimes beyond reclamation, with the balance in a perfectly normal condition. It is my observation that, as before mentioned, the trouble in these cases is most usually at the interdental and palatal aspects of the superior molars, and lingual, mesial and distal aspects of the lower molars, a strong argument for the contention of the adherents of the local cause theory—because these are the surfaces that collect and retain the debris of the mouth with most tenacity, the most difficult to clean, and in the mouth of most people receive practically no attention at all. I have seen a number of these cases with the molars positively past redemption, while the tissues surrounding the other teeth were in perfect condition.

In my locality, fortunately pyorrhea is not so prevalent as it seems to be in many others. Nor does it seem to generally affect all the teeth in the mouth, but the usual manifestation that we see presents only a moderate inflammation and turgidity of the gums—in fact so slight that one would hardly suspect a pocket—no pus—seruminal deposit sometimes absent, but usually enough to give that "sandy" feeling to the instrument which we all recognize so well, but with pockets deeper and more extensive than the usual pus pockets. Now these are the manifestations, to my mind, presenting the most unfavorable prognosis, for while it is possible to treat and reduce the inflammation and turgidity, and restore the gum to its normal condition, it seldom reattaches itself to the tooth, remaining for all time thoroughly detached, susceptible at all times to a reoccurrence of the original conditions and requiring close attention constantly.

Are you familiar with the characteristic odor of pyorrhea? What an odor, some one has likened it to the smell of rotten cabbage, and I know of nothing that describes it quite so well, but I have noticed in all cases, that this, with the constant bleeding of the gums may be completely relieved by thorough local treatment.

Another manifestation that may puzzle the uninitiated is the pericemental abscesses which we often encounter; the pain is intense, but unless the cause is beyond reach, as is sometimes found in the molars, or the tooth is beyond reclamation, they are usually amenable to treatment.

Now, to use the time honored query, "What are you going to do about it?" Do you belong to that class who have been told, and believe that pyorrhea is incurable? If so, retrace your steps and join hands with the constructive element of your profession who believe it their duty to overcome obstacles, and add to the comfort and happiness of mankind. You may not be successful in all your efforts, but will be so in sufficient measure to amply reward you.

Before we mention the means for the relief and cure of this condition let me say in parenthesis that I am thinking both from the standpoint of operator and patient, as I have tackled both ends of the proposition.

As before mentioned, the belief in the constitutional origin of this disease is pretty generally accepted, and it would seem that the first step should look to the removal of the cause, and so it should, but as far as the general practitioner is concerned, experience has shown that the means to this end are to only a very slight extent under his control. The evils of auto intoxication are legion and severe, but it is functional and not organic, and so, while remedies and medication have been suggested *ad infinitum*, they are all only of superficial and temporary value, and the only real relief lies in a complete and thorough hygenic way of living. Temperance in all things, particularly in eating and drinking, change of environment, out door living, rest and recreation, for the overworked, work for the idle, and for all, clean and wholesom habits and thoughts. You seldom find pyorrhea among people who live an active out door life and are half way observant of the simplest hygenic precautions. So you see, that as far as the constitutional conditions are concerned it is a matter that is far from the dentists control. Drugs won't do the work.

Let me say right here that I have treated many beer and whisky guzzlers, but beyond superficial and temporary results, have never been able to make much of an impression. I don't believe a man who keeps his hide soaked with whisky can be cured of anything. Certainly not of pyorrhea.

The constitutional treatment of pyorrhea by the use of vaccines raising the opsonic index, promises much, and in my opinion is the next big thing coming to dentistry, in fact we may say it "has arrived," but at present, has not been made available for the use of the general practitioner.

As to the local treatment: now we come to the meat of the matter, as far as we are concerned. As before stated there seems to be little doubt that pyorrhea is the local manifestation of a functional and constitutional trouble, but the local manifestation may be controlled and held in abeyance by local means, and this is what we mean, at present, by curing pyorrhea. Now another point upon which all are agreed, namely: When a case of pyorrhea is once cured it requires the most constant and scrupulous care on the part of the patient, with regular periodical care by the dentist to keep it cured. We can not hope to restore any lost tissue—or to remove the susceptibility—all we can hope for is to stop the progress of the disease.

We shall not attempt a detailed description of the technique in the local treatment, as it, with the prophylaxis to be continued after the surgical treatment has been described so often, and, no doubt, will be many times during this meeting, that it is not necessary, but will content myself with a few specific observations.

All workers are agreed that the keynote of success lies primarily and unquestionably in the surgical procedures and the thorough removal of all deposits and necrosed tissue. There certainly can not be success without this.

So the training of the sense of touch to appreciate the feel of deposits, and the feel of clean tooth surfaces that are removed from the field of vision, and with an ability to hunt out all obscure nooks and angles, constitute the first thing for the general practitioner to learn and appreciate.

The idea seems to be to thoroughly scale every surface of the root, remove every vestige of anything foreign, all necrotic edges of alveolar process, "*and then some.*" My suggestion is that you do it thoroughly and then stop, because it is nearly always live tissue you are operating upon. Pyorrhea and sound teeth usually go together—not always, but usually, and there are after effects to be reckoned with. (I am patient as well as doctor you know. It gives me a chill even now to think of a drink of cold water and starts the perspiration to think of a piece of candy)

and then "enough of anything is enough, and too much is more than a plenty." I have heard of operators devoting one or two hours to the treatment of a single tooth, such a thing to my mind is inconceivable, as I see no reason why all that can be done should not be done in a reasonable length of time, but the idea of thoroughness must not be lost sight of under any circumstances.

Another point, or rather two points, upon which all are agreed; that all teeth which are evidently beyond saving should be extracted and replaced by bridge work of some kind, if feasible.

To be able to determine just when a tooth can not be saved is a valuable attainment and can only be acquired by experience, as it is certain that when the ravages of the disease have progressed to a certain stage nothing can be done, and it is a mistake to make the effort without a reasonable hope of success.

Instrumentation.—In the proper surgical treatment much depends upon the instruments used. Instruments for this purpose have been devised without limit, many that are good, and more that are worthless, but I would like to state most emphatically that no set consisting of 117 or 192 instruments is necessary, or even permissible, as complicated curves and angles and flexible shanks are fatal mistakes. Complicated angles and curves are fatal to the delicate sense of touch necessary, and the flexibility of the long shank permits the blade to spring and slip over the deposits without removing them.

In the earlier development of the treatment of pyorrhea the instruments of that character to be used with a push motion were used almost exclusively, but I believe that those to be used with the pulling motion are now generally accepted.

There are two good reasons for this, the first and strongest is that instruments of this character are calculated to draw out and remove the deposits when they are detached, which is a matter of some consideration, while the effect of the push instruments is just the opposite.

The second one is that there is less unnecessary laceration of the soft tissues, and all are agreed that this should be reduced to a minimum. This caries with it, of course, the fact that it is less painful to the patient—and this is no mean consideration.

I have tried pretty near all the instruments I have ever seen, but have settled down to the use of what is known as the D. D. Smith instruments, those having a number of blades on a flat surface after the manner of a coarse file. These instruments have the effect of breaking up the serumnal deposits into small particles when drawn over them, and removing them as such, just as Dr. Arrington, of North Carolina, used to advocate the breaking up of these deposits with a burnisher, and then they leave the root smoother than any other instrument that I know of. Dr. Towner has a modification of these instruments which are also valuable.

I find that about six, having good strong shanks, with the simplest curves and angles, in addition to a few ordinary scalers for use on exposed surfaces are about all I need. Of course, as to the question of instruments the factor of personal equation operates to a greater extent than most any other, and one must be governed largely by what is most efficient in his own hands. It is pretty generally observed that the more manual dexterity one acquires the fewer and the simpler the instruments required.

Now as to the drugs and local applications used in the treatment, let me say at the outset, don't be deceived, turn a deaf ear to the dealer who proposes to sell you a specific and guaranteed cure for pyorrhea at so much per—as this is a mere "will o' the wisp"—no drug or medicament has ever yet been suggested that is anything more than a secondary matter, the various antiseptics and astringents suggested perhaps have a value, but as before stated all hopes for success must be based upon thorough surgical treatment. I have never thought to try it, but I believe as good results can be obtained by the liberal use of tepid water as anything else. I believe Dr. Patterson says, however, that he believes more prompt results may be obtained by the use of some good germicidal astringent, used in the pockets. Of course the use of peroxide of hydrogen is to be recommended for its faculty of cleansing the pockets of debris and blood clots. The use of solvents that has been advocated by many I believe to be unnecessary at best, and even harmful—I refer to bi-flouride of amonia, sulphuric acid, lactic acid and agents of a like nature, neither do I believe the escharotic germicides, such as carbolic acid are indicated, but believe that only mild germicidal astringents should be used.

I have used nearly everything that has been mentioned, but I began with the Iodol-glycerol preparation suggested by Dr. Talbot and have returned to it and am making it my sole remedy at present.

There is nothing better for the gums, the pockets and the roots than a 10 percent. solution of nitrate of silver.

If you are in a hurry and can not remember the proportions of something you have heard recommended, just take equalparts of iodine and old-fashioned turpentine and add enough alcohol to cause themto combine, and I believe you will have as good a remedy as any other. Let me emphasize the importance of throough prophylaxis to be observed during the treatment andto be maintained for all time afterward, as no case of pyorrhea will stay cured without it. I shall not describe this as it is familiar to all.

Before closing, let me mention, just briefly, three things that I wish to emphasize.

(1) If you think of using the D. D. Smith instruments be very careful not to break one of them off in a deep rigid pocket, as there is apt to be extreme difficulty in removing it.

(2) Beware of overtreatment, as healing tissue should not be disturbed, and nature should have a square deal—I have hinted at this before, and it applies to all pathological conditions that I know anything about.

(3) Do not attempt to polish with wood points and pumice that portion of the root beyond the gum margin. You can not do it thoroughly anyway, and if you attempt it, the injury from the laceration of the gums will prove a positive disadvantage rather than a help.

